# The splicing factor SRSF3 is functionally connected to the nuclear RNA exosome for intronless mRNA decay

**DOI:** 10.1038/s41598-018-31078-1

**Published:** 2018-08-27

**Authors:** Fabrice Mure, Antoine Corbin, Nour El Houda Benbahouche, Edouard Bertrand, Evelyne Manet, Henri Gruffat

**Affiliations:** 1 CIRI, Centre International de Recherche en Infectiologie, (Oncogenic Herpesviruses Team), Univ Lyon; Inserm, U1111, Université Claude Bernard Lyon 1; CNRS, UMR5308; ENS de Lyon, F-69007 Lyon, France; 2 Equipe labellisée Ligue contre le Cancer, Institut de Génétique Moléculaire de Montpellier (IGMM), Université de Montpellier; CNRS, UMR5535, F-34293 Montpellier, France

## Abstract

The RNA exosome fulfills important functions in the processing and degradation of numerous RNAs species. However, the mechanisms of recruitment to its various nuclear substrates are poorly understood. Using Epstein-Barr virus mRNAs as a model, we have discovered a novel function for the splicing factor SRSF3 in the quality control of nuclear mRNAs. We have found that viral mRNAs generated from intronless genes are particularly unstable due to their degradation by the nuclear RNA exosome. This effect is counteracted by the viral RNA-binding protein EB2 which stabilizes these mRNAs in the nucleus and stimulates both their export to the cytoplasm and their translation. In the absence of EB2, SRSF3 participates in the destabilization of these viral RNAs by interacting with both the RNA exosome and its adaptor complex NEXT. Taken together, our results provide direct evidence for a connection between the splicing machinery and mRNA decay mediated by the RNA exosome. Our results suggest that SRSF3 aids the nuclear RNA exosome and the NEXT complex in the recognition and degradation of certain mRNAs.

## Introduction

In eukaryotic cells, functional mRNA expression is a multi-step process in which the DNA-encoded message is transcribed into a pre-mRNA molecule that undergoes numerous modifications such as 5′-end capping, splicing, 3′-end cleavage and polyadenylation, together with the assembly of diverse factors required for the formation of a messenger ribonucleoprotein particle (mRNP)^1,2^. The adequately processed mRNPs are then competent for their export to the cytoplasm where they will be translated^3^. All these processes are intimately linked: 5′-end capping, splicing and 3′-end maturation occur co-transcriptionally due to the important role played by the carboxy-terminal domain (CTD) of RNA polymerase II’s (RNAP-II) largest subunit^4,5^. However, mRNA processing is error-prone and improperly matured mRNPs have to be degraded in order to avoid the synthesis of nonfunctional proteins. While the synthesis of the mRNPs progresses, surveillance mechanisms that detect malformed mRNPs are also operating. Aberrant mRNPs^6^ that fail to pass the quality control steps are retained in the nucleus and degraded by different ribonucleases. In human cells, two major degradation pathways are responsible for mRNA decay of defective transcripts in the nucleus: (i) the 5′-3′ exoribonuclease XRN2, together with the decapping factor DCP2, and (ii) the RNA exosome^7,8^.

The RNA exosome complex, first described in yeast, is conserved in all eukaryotic cells. In human cells, it is composed of a core of nine subunits which serves as a binding platform for two active ribonucleases - hRRP6 and hDIS3/hRRP44 - that have 3′-5′ RNA exonuclease and endonuclease activities^9,10^. This complex recognizes and degrades improperly-formed RNAs in the nucleus^11^. To exert its function, the nuclear RNA exosome uses cofactors that directly stimulate its enzymatic activity and serve as adaptors for its many substrates^12^. Several proteins or complexes have recently been identified for their capacity to recruit the nuclear RNA exosome onto its target RNAs. In the yeast system for which several exosome-associated adaptors have been characterized, it appears that the nuclear RNA exosome depends largely on the activities of the TRAMP (Trf4p/5p-Air1p/2p-Mtr4p polyadenylation) complex^13–19^. However, in human, at least three distinct RNA exosome adaptors have recently been identified. All critically depends on the RNA helicase hMTR4: the hTRAMP complex, which is homologous to the yeast complex and localizes in the nucleolus^20–22^, the PAXT (poly(A) tail exosome targeting) complex formed by hMTR4-ZFC3H1 and the NEXT (nuclear exosome targeting) complex which is not conserved in yeast and localizes in the nucleoplasm^21,23–25^. The RBM7 protein, a putative pre-mRNA splicing factor, and the ZCCHC8 (zinc finger CCHC domain-containing protein 8) protein form the NEXT complex. Interestingly, ZCCHC8 has also been shown to interact with the cap-binding complex (CBC) and several members of the SR protein family^21^, and one study has reported that, *in vitro*, RBM7 interacts with the splicing factor 3b subunit 2 (SAP145) and the SRSF3 protein^26^. The interaction between SRSF3 and RBM7 was also found by a proteomic approach^27^. This suggests that the cap-binding complex and the spliceosome are involved in the recruitment of the RNA exosome to its target RNAs via their interaction with the NEXT complex. Moreover, it was recently demonstrated that the recruitment of the RNA exosome by CBC promotes the degradation of promoter upstream transcripts (PROMPTs)^27^. The role of the spliceosome in mRNA decay is also suspected because an interaction has been found between hMTR4 and hRRP6 and several components of the spliceosomal U4/U6.U5 tri-snRNP complex^28^. However, a direct functional link between the splicing reaction and the mRNA decay machinery is lacking.

Epstein-Barr virus (EBV), a ubiquitous human herpes virus, is the causative agent of infectious mononucleosis and is also associated with various cancers observed in immuno-competent individuals (Burkitt’s lymphoma, Hodgkin’s disease, nasopharyngeal carcinoma, T/NK lymphoma, and a subtype of gastric carcinoma) as well as in immuno-deficient patients (lymphoma and post-transplant lympho-proliferative diseases)^29^. EBV persists in an infected host for life by establishing a latent infection in memory B cells^30^. Following cell differentiation, viral reactivation into the productive cycle leads to the production of progeny viruses that spread to new cells and hosts^31^. For these two phases of the viral life cycle different viral gene expression programs are used. During latency, a small subset of EBV genes is expressed that allows maintenance of the viral genome and induction of cell proliferation and differentiation in the absence of viral production. Genes expressed during this phase have the same structure as cellular genes. By contrast, during the productive cycle, the structure of a majority of the viral genes expressed during this phase differs from that of cellular genes because most of the viral genes are intronless. This characteristic may represent an handicap for the viral mRNAs in terms of cytoplasmic accumulation and translation efficiency since it is now well documented that pre-mRNA splicing efficiently increases both mRNA export and translation^3,32,33^. However, EBV - like all herpesviruses - encodes a protein called EB2 (also referred to as BMLF1 or SM) whose role is to increase the cytoplasmic accumulation and translation of viral mRNAs expressed from intronless genes^34–38^. EB2 is an RNA-binding protein (RBP)^39^ essential for virus replication^40^ that shuttles between the nucleus and the cytoplasm^41,42^. It has been reported to facilitate the nuclear export of viral mRNAs expressed from intronless genes by interacting with the cellular ALYREF and TAP-NXF1/p15 proteins that promote cellular mRNA export^41,42^. Thus, EB2 may serve as a bridge between viral RNAs and the cellular export complex (TREX), which is recruited to the 5´-end of cellular RNAs as a result of splicing^43^. In this model, EB2, like homologous proteins from other herpesviruses, compensates for the absence of splicing of the viral mRNAs expressed from intronless genes, by recruiting the TREX complex. Accordingly, EB2 is dispensable for mRNAs expressed from intron-containing genes. In addition, EB2 is able to inhibit the splicing reaction^35,44^ and modify the splicing pattern of specific cellular mRNAs^45^. The interaction observed between EB2 and SR proteins like SRSF1 (ASF/SF2), SRSF3 (SRp20) and SRSF7 (9G8) is probably involved both in the modifications of specific cellular genes splicing and in the increase in nuclear mRNA export of viral mRNA expressed from intronless genes^46–48^. Interestingly, EB2, like its homologous protein ORF57 from Kaposi’s sarcoma-associated herpesvirus, has also been suggested to be involved in increasing mRNA stability. However, the mechanisms involved are not yet understood^49,50^.

In the present study, we show that in addition to its well-documented role in mRNA export and translation, the viral RNA-binding protein EB2 is also involved in mRNA stability control. In the absence of EB2, its target mRNAs - produced from intronless genes - are rapidly degraded by the nuclear RNA exosome. We have identified the splicing factor SRSF3 as being responsible for the destabilization of these viral mRNAs. Our data reveal, for the first time, that SRSF3 associates with both the NEXT complex and the RNA exosome to induce degradation of mRNAs in the nucleus. The viral protein EB2 appears to interfere with this processus, thus rescuing viral mRNAs from degradation. Our findings reveal an important novel function of the SR protein SRSF3, in the recognition and degradation of some mRNAs.

## Results

### The EB2 protein is not only an mRNA export factor

**Figure 1:**
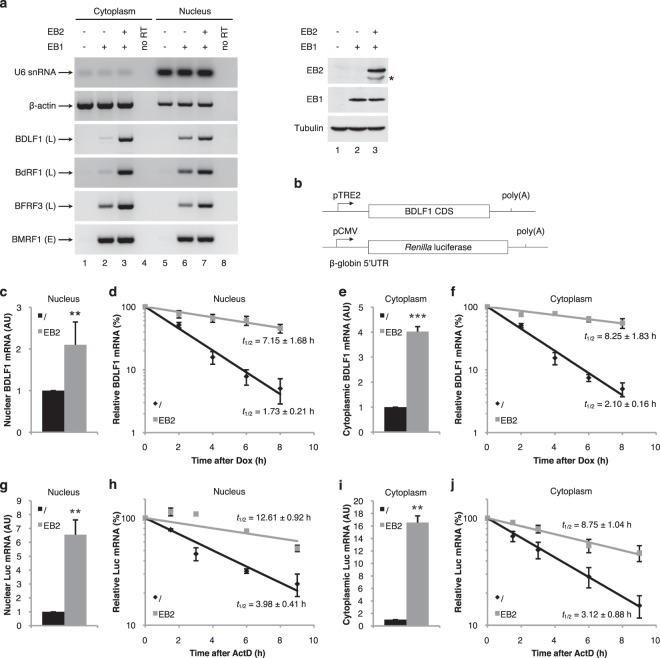
EB2 stabilizes late viral mRNAs in the nucleus. (**a**) *mRNA accumulation:* Cytoplasmic and nuclear RNAs from HEK293_EBV∆BMLF1_ cells transiently transfected as indicated at the top of the figure were submitted to RT-PCR analysis using specific primers to detect cellular U6 snRNA and ß-actin mRNA, or EBV-encoding mRNAs (BDLF1, BdRF1, BFRF3 and BMRF1). The PCR products were loaded on a 2% agarose gel and visualized by ethidium bromide staining. The RT-PCR results were in the linear range of the PCR reaction. Expression of EB2, EB1 and Tubulin proteins expressed in HEK293_EBV∆BMLF1_ cells that have been transfected, or not (lane 1), with an EB1 expression plasmid (lane 2), or cotransfected with expression plasmids for both EB1 and EB2 (lane 3) were controlled by western-blotting. * Indicates an unspecific band recognized by the anti-EB2 serum. (**b**) Schematic representation of the pTRE2-BDLF1 construct which contains the viral gene BDLF1 under the control of the Tet-responsive promoter and the pCMV-RLuc construct which contains the Renilla Luciferase gene, RLuc, under the control of the CMV promoter. (**c**) (**e**) (**g**) and (**i**) *Nuclear and cytoplasmic BDLF1and luciferase mRNAs accumulation:* Quantification by RT-qPCR of the nuclear (**c** and **g**) and cytoplasmic (**e** and **i**) BDLF1 and luciferase mRNA expressed from HeLa cells co-transfected with the pTRE2-BDLF1 construct or the pCMV-RLuc construct without or with an EB2 expression vector (time 0 of the kinetic). Error bars represent the s.d. from three independent experiments (*n* = 3). ** Indicates *P*-value of <0.01 and *** indicates *P*-value of <0.001. (**d**) (**f**) (**h**) and (**j**) *Nuclear and cytoplasmic BDLF1 or luciferase mRNA stability:* Relative nuclear (**d** and **h**) and cytoplasmic (**f** and **j**) BDLF1 or luciferase mRNA level after transcription inhibition (expressed as a percentage of the amount at time point 0) was determined by RT-qPCR from HeLa cells co-transfected with the pTRE2-BDLF1 construct or the pCMV-RLuc construct without or with an EB2 expression vector. Numbers above the panel refer to hours after transcriptional shutoff of the BDLF1 or luciferase intronless gene. Half-lives (*t*_1/2_) and s.d were calculated from three independent experiments (*n* = 3).

It has been extensively shown that the viral protein EB2, as well as its homologs from other herpesviruses - ICP27, UL69 and ORF57 - are involved in the cytoplasmic accumulation of viral mRNAs expressed from intronless genes. That EB2 also has a function in nuclear stabilization of its target mRNAs was suggested by the observation that the EB2’s target mRNAs are not well accumulated in the cell nucleus in the absence of EB2^49^. To explore this further, cytoplasmic and nuclear RNAs were purified from HEK293_EBV∆BMLF1_ cells^40^ which contain a recombinant virus defective for the expression of EB2. The relative amounts of several viral mRNAs whose cytoplasmic accumulation was known to be dependent on EB2 (BDLF1, BdRF1 and BFRF3) or not (BMRF1) was then measured, by RT-PCR (Fig. 1a). U6 snRNA and ß-actin mRNA were used as controls for the purification efficiency of the nuclear and cytoplasmic mRNAs, respectively. As expected, in the absence of viral reactivation, the viral early (BMRF1) and late (BDLF1, BdRF1 and BFRF3) mRNAs were not expressed. When the viral productive cycle was activated by ectopic expression of the viral transcription factor EB1, the late mRNAs known to be targeted by EB2 were barely detected in the cell cytoplasm but accumulated in the cell nucleus. This nuclear accumulation argues in favor of a role for EB2 in viral mRNA export as has already been reported^34^. However, when EB2 was expressed in the cells, EB2-dependent viral mRNAs not only accumulated in the cytoplasm as a result of EB2-mediated mRNA export, but their expression was also clearly enhanced in the nucleus. These results suggest that EB2 could play a role in nuclear mRNA stability.

To test this hypothesis, the nuclear stability of an EB2 target mRNA, BDLF1, and a Luciferase reporter mRNA, were compared, in the presence or absence of the EB2 protein. For this, HeLa cells were transfected with the pTRE2-BDLF1 reporter plasmid in which BDLF1’s transcription is under the control of doxycycline or the pCMV-Renilla Luciferase reporter plasmid (Fig. 1b), together, or not, with an EB2 expression plasmid. 24 h after transfection, the cells were treated either with doxycycline in order to stop transcription of the BDLF1 reporter gene, or with actinomycin D to stop transcription of the Luciferase gene, and both nuclear and cytoplasmic mRNA, purified at different time points, were quantified by RT-qPCR. As expected, at time zero, the BDLF1 and the Luciferase mRNA levels were more important in both the nucleus and the cytoplasm of cells transfected with an EB2 expression vector than in the control cells (Fig. 1c,e,g,i). Quantification of BDLF1 and Luciferase mRNAs during the time-course following inhibition of their transcription, revealed that they were much more stable in the nucleus of cells expressing the EB2 protein (Fig. 1d,h). In the presence of EB2, the half-life of the reporter mRNAs was increased over four-fold. Interestingly, the half-life of cytoplasmic reporter mRNAs was very similar to that observed for the nuclear mRNA, both in the absence or presence of EB2 (Fig. 1f,j). This observation suggests that nuclear export is not likely to be responsible for the reporter mRNA half-life changes observed in the different conditions. Taken together, these results clearly demonstrate that EB2 enhances nuclear mRNA stability of specific transcripts.

### The nuclear RNA exosome is involved in the decay of EB2’s target mRNAs

**Figure 2:**
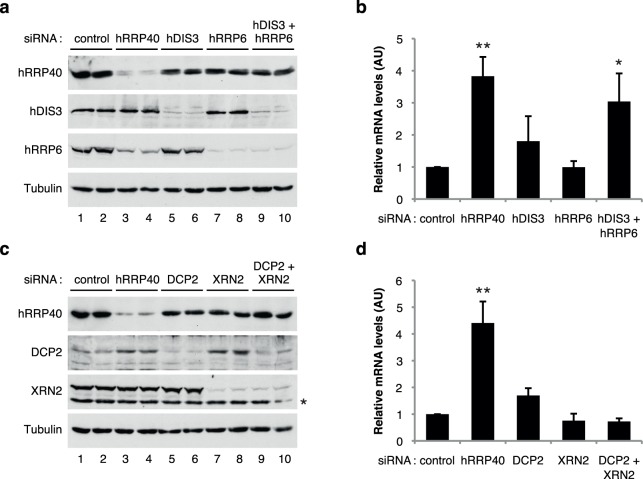
EB2’s target mRNAs are mainly degraded by the nuclear RNA exosome. (**a**) and (**c**) *Immunoblot:* Western blotting analysis of cell extracts showing protein depletion upon the indicated siRNA treatments. Hela cells were treated with specific or control (Firefly) siRNAs. Membranes were probed with the indicated antibodies (anti-hRRP40, -hDIS3, -hRRP6, -DCP2, -XRN2 and -Tubulin). Anti-Tubulin antibody was used as loading control. * Indicates non-specific bands. (**b**) and (**d**) *Nuclear mRNA accumulation:* RT-qPCR analysis of nuclear RNAs expressed from HeLa cells transfected with the indicated siRNAs and the Renilla luciferase intronless gene. Data are displayed as mean values normalized to the control siRNA and GAPDH mRNA as an internal control. Error bars represent the s.d. from three independent experiments (*n* = 3). * Indicates a *P*-value of <0.05 and ** indicates a *P*-value of <0.01.

Eukaryotic mRNAs contain two features important for their stability: the 5′ 7-methylguanosine cap and the 3′ poly(A) tail. In the cell nucleus, these determinants interact respectively with the cap-binding complex (CBC) and the nuclear poly(A)-binding protein (PABPN1) to protect the transcripts from exoribonucleases and to promote the export of the mRNAs. To initiate mRNA decay, either one of these two structures must be compromised or the mRNA must be cleaved by endonucleolytic attack. Two main pathways have been described for the degradation of nuclear mRNAs: either the 5′cap structure is removed by the decapping enzyme DCP2 thus allowing the mRNA body to be degraded in the 5′-3′ direction by the XRN2 exoribonuclease or the 3′ poly(A) tail is attacked by a large exonuclease complex known as the RNA exosome^51^. In order to determine which of these pathways is responsible for the nuclear decay of intronless mRNAs, expression of specific components of these different machineries was down-regulated using siRNAs. Depletion efficiency of the proteins was determined by western blotting (Fig. 2a,c). Interestingly, down-regulation of hRRP40, a component of the core RNA exosome complex, induced a net stabilization of the Renilla luciferase reporter mRNA (Fig. 2b,d). Down-regulation of either of the two ribonucleases associated with the RNA exosome core complex resulted in only a weak effect (hDIS3/hRRP44) or the absence (hRRP6) of an effect on the RLuc reporter mRNA stability. However, simultaneous down-regulation of the two ribonucleases enhanced the stability of the target mRNA to a level similar to what was seen when hRRP40, the core component of the RNA exosome, was down-regulated (Fig. 2b). Taken together, these results indicate first, that the RNA exosome pathway is important for decay of EB2’s target mRNAs, and second, that both ribonucleases associated with the RNA exosome core complex are involved in the degradation pathway.

By contrast, when the 5′-3′ decay pathway was down-regulated by siRNAs directed against either DCP2 or XRN2 proteins or both, the stability of the RLuc reporter mRNA was not significally altered compared to what was observed in the case of down-regulation of the RNA exosome pathway (Fig. 2d). Thus, nuclear decay of EB2’s target mRNAs appears to depend mainly on the action of the nuclear RNA exosome.

### The nuclear RNA exosome impacts EBV viral gene expression and virus production

**Figure 3:**
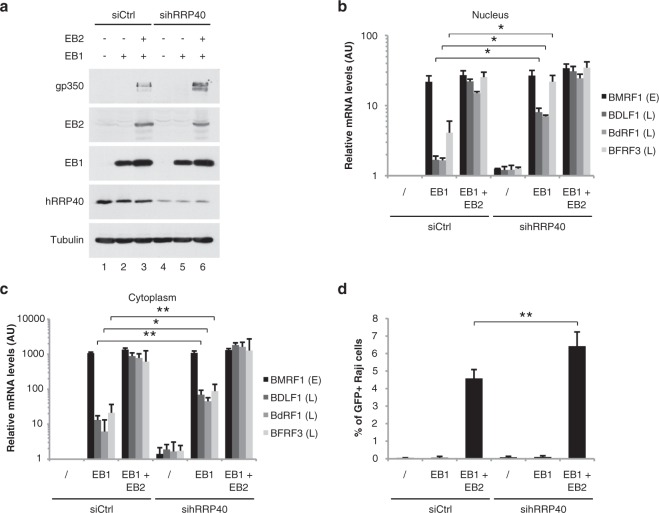
RNA exosome depletion increases both nuclear accumulation of viral intronless mRNAs and virion production. (**a**) *Immunoblot:* Western blotting analysis of HEK293_EBV∆BMLF1_ cells extracts showing protein depletion upon hRRP40 siRNA treatments. HEK293_EBV∆BMLF1_ cells were treated with specific (hRRP40) or control (Firefly) siRNAs and transfected with a control vector (lanes 1 and 4) or an EB1 expression vector in order to induce the viral productive cycle (lanes 2, 3, 4 and 5) and transcomplemented with an EB2 expression vector (lanes 3 and 5). Membranes were probed with the indicated antibodies (anti-hRRP40, -EB1, -EB2, -gp350 and -Tubulin). Anti-Tubulin antibody was used as a loading control. (**b**) and (**c**) *Nuclear and cytoplasmic mRNA accumulation:* Quantification by RT-qPCR of nuclear (**b**) and cytoplasmic (**c**) EBV-encoding early (E) mRNA (BMRF1) or late (L) mRNAs (BDLF1, BdRF1 and BFRF3) in HEK293_EBV∆BMLF1_ cells transfected as described in (**a**). Data are displayed as mean values normalized to the control siRNA and GAPDH mRNA as an internal control. Error bars represent the s.d. from three independent experiments (*n* = 3). * Indicates a *P*-value of <0.05 and ** indicates a *P*-value of <0.01. (**d**) *Virus production:* HEK293_EBV∆BMLF1_ cells supernatant were collected 48 h after the induction of the productive cycle, filtered and used to infect Raji cells. The number of EBV-infected Raji cells was evaluated by quantification of GFP-expressing cells 72 h later by FACS analysis. Error bars represent the s.d. from three independent experiments (*n* = 3). ** Indicates a *P*-value of <0.01.

Since RNA exosome depletion has an important effect on the nuclear stability of RLuc intronless mRNA, we asked whether depletion of hRRP40 (one of the core RNA exosome subunits) could also affect the EB2-dependent nuclear accumulation of specific viral mRNAs as well as viral production. For this, expression of the nuclear RNA exosome was down-regulated in the HEK293_EBV∆BMLF1_ cell line, by transfection of a siRNA specifically directed against hRRP40 (Fig. 3a). 48 h after activation of the viral productive cycle, the viral nuclear and cytoplasmic mRNAs were quantified by RT-qPCR, and viruses produced in the cell culture supernatant were titrated. Interestingly, in the absence of EB2, depletion of the RNA exosome allowed a net nuclear accumulation of the late viral mRNAs BDLF1, BdRF1 and BFRF3, whereas the amount of the BMRF1 early mRNA was not affected (Fig. 3b). By contrast, in the presence of EB2, depletion of the RNA exosome had only a weak but reproducible effect on late viral nuclear mRNA accumulation. It is interesting to note that these results are very similar to what was observed for the cytoplasmic accumulation of viral mRNAs in cells from which SRSF3 had been depleted (Fig. 3c) as already reported^47^. As expected from the cumulative effect on the stabilization of different viral and cellular mRNAs following RNA exosome depletion, virus production was reproducibly higher from cells trans-complemented with EB1 and EB2, when hRRP40 was down-regulated (Fig. 3d). However, in the absence of EB2, hRRP40 depletion was clearly not sufficient to allow virus production even though this depletion enhances the nuclear and cytoplasmic accumuation of the late viral mRNAs as shown above in Fig. 3b and c. This absence of virus production could be easily explained by the fact that EB2 is also involved in mRNA export and translation^36,52^. Taken together, these results suggest that in the absence of the viral protein EB2, the nuclear RNA exosome induces mRNA decay of some viral mRNAs. However, the presence of EB2 counteracts this negative effect of the RNA exosome on these intronless mRNAs.

### SRSF3 is involved in viral mRNA stability

**Figure 4:**
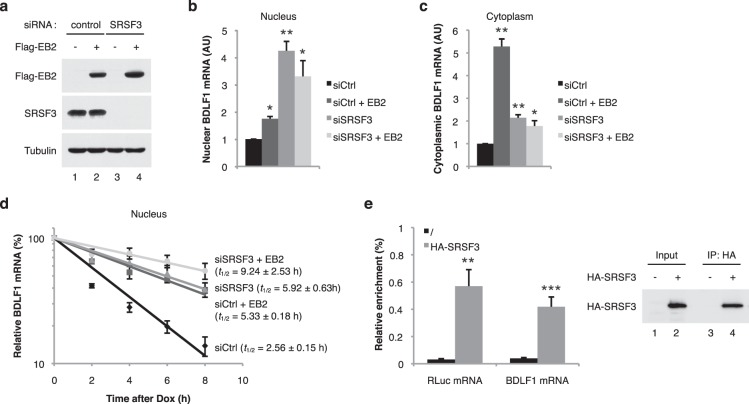
Depletion of the splicing factor SRSF3 stabilizes intronless mRNAs in the nucleus. (**a**) *Immunoblot:* Western blotting analysis of HeLa cells transfected with the pTRE2-BDLF1 construct without or with an EB2 expression plasmid as indicated. Cells were previously transfected with either a control siRNA (lanes 1 and 2) or a siRNA specific for SRSF3 (lanes 3 and 4). The western blots were probed with either an anti-Flag antibody to detect Flag-tagged EB2, an anti-SRSF3 or an anti-Tubulin antibody as a loading control. (**b**) and (**c**) *Nuclear and cytoplasmic BDLF1 mRNA accumulation:* Quantification by RT-qPCR of nuclear (**b**) and cytoplasmic (**c**) BDLF1 mRNA from HeLa cells transfected as described in (**a**). Error bars represent the s.d. from three independent experiments (*n* = 3). * Indicates a *P*-value of <0.05 and ** indicates a *P*-value of <0.01. (**d**) *Nuclear BDLF1 mRNA stability:* Relative nuclear BDLF1 mRNA level after transcription inhibition (as a percentage of the amount at time point 0) was determined by RT-qPCR from HeLa cells transfected as described in (**a**). Numbers above the panel refer to hours after doxycycline-mediated transcriptional shutoff of the BDLF1 intronless gene. Half-lives (*t*_1/2_) and s.d were calculated from three independent experiments (*n* = 3). (**e**) *RNA immunoprecipitation:* HeLa cells were transfected with the pCMV-BDLF1 or the pCMV-RLuc reporter constructs together with a HA-SRSF3 expression plasmid as indicated. SRSF3 containing complexes were immunoprecipitated using an anti-HA antibody. RNA was then purified and the amount of specific BDLF1 and RLuc transcripts co-immunoprecipitated was evaluated by RT-qPCR. The amount of SRSF3 expressed and immunoprecipitated in the samples was evaluated by western blot analysis. Error bars represent the s.d. from two independent experiments (*n* = 2). * Indicates a *P*-value of <0.05 and ** indicates a *P*-value of <0.01.

Interaction between EB2 and the splicing machinery has been previously documented^46,47^ and interestingly, we recently found that SRSF3 (SRp20) depletion results in an increase in the cytoplasmic accumulation of EB2’s target mRNAs^47^. This suggests that EB2 functions by antagonizing SRSF3 and that the splicing factor SRSF3 might affect mRNA stability. To investigate this possibility, the pTRE2-BDLF1 reporter plasmid was transfected into HeLa cells previously depleted of SRSF3 by siRNA treatment (Fig. 4a). The amount of nuclear and cytoplasmic BDLF1 mRNA expressed was then quantified by RT-qPCR (Fig. 4b,c) and its nuclear stability assessed after addition of doxycycline (Fig. 4d). As shown in Fig. 4b,c, EB2 increased the nuclear and cytoplasmic accumulation of BDLF1 mRNA. Down-regulation of SRSF3 also led to an accumulation of nuclear and cytoplasmic BDLF1 mRNA and this accumulation was slightly affected by co-expression of EB2. However, the cytoplasmic accumulation was less important than the nuclear accumulation because as we have previously shown^47^, SRSF3 is required for the cytoplasmic export of this intronless mRNA. This combined effect of EB2 and SRSF3 could be the consequence of either the presence of residual SRSF3 in cells treated with the siRNA directed against SRSF3 or a slight increase in the expression of EB2 as detected by western blot (Fig. 4a, lane 4) or both. During the time-course study, it appeared that down-regulation of SRSF3 stabilized the BDLF1 mRNA in the nucleus to a level similar to that seen when EB2 was expressed in the cells (Fig. 4d). These results suggest that EB2 antagonizes the destabilizing effect of SRSF3 on nuclear BDLF1 mRNA. SRSF3 plays an important role in the metabolism of cellular mRNA, especially intronless mRNA. It has been shown that SRSF3 has to be recruited onto the target mRNA to have an effect^47,48^. In order to demonstrate that SRSF3 is recruited onto its target mRNA, HeLa cells were transfected with a HA-SRSF3 expression vector together with the BDLF1 or Luciferase reporter plasmids. 48 h later, the SRSF3 protein was immunoprecipitated and the presence of the BDLF1 or Luciferase mRNA associated with SRSF3 was analysed by RT-qPCR (Fig. 4e). The results show that both the BDLF1 and the Luciferase mRNA specifically interact with SRSF3. Taken together, these data suggest that SRSF3, when bound onto its target mRNAs, recruits a complex involved in mRNA degradation.

### The splicing factor SRSF3 and the RNA exosome complex are linked in the nuclear mRNA decay of unspliced mRNA

**Figure 5:**
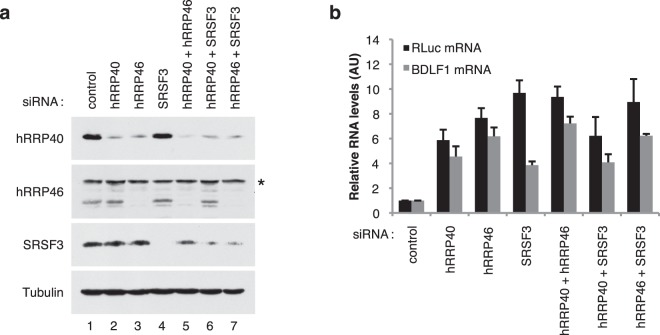
Intronless mRNAs do not hyperaccumulate in the nucleus upon co-depletion of the RNA exosome and the SRSF3 protein. (**a**) *Immunoblot:* Western blotting analysis showing protein depletion upon the indicated siRNA treatments. Anti-Tubulin antibody was used as a loading control. * indicates non-specific bands. (**b**) *Nuclear mRNA accumulation:* RT-qPCR analysis of nuclear RNAs from HeLa cells transfected with the indicated siRNAs together with the Renilla luciferase and BDLF1 intronless constructs. Data are displayed as mean values normalized to the control siRNA and GAPDH mRNA as an internal control. Error bars represent the s.d. from three independent experiments (*n* = 3).

Down-regulation of SRSF3 expression in the cells leads to the stabilization of nuclear mRNAs whose expression are dependent on EB2. Furthermore, we have previously shown that the nuclear RNA exosome pathway is the major contributor to the nuclear mRNA decay observed for EB2’s target mRNAs. In order to determine whether SRSF3 and the nuclear RNA exosome function (or not) within the same pathway, the Renilla luciferase and BDLF1 reporter plasmids were transfected into HeLa cells from which the RNA exosome (hRRP40 or hRRP46 core components) or/and SRSF3 were depleted (Fig. 5a). The nuclear accumulation of the reporter mRNA was then measured 24 h post-transfection (Fig. 5b). As expected, depletion of either the nuclear RNA exosome or SRSF3, alone, resulted in an increased accumulation of the reporter mRNAs. However, co-depletion of either SRSF3 and hRRP40 or SRSF3 and hRRP46, did not further increase the amount of accumulated nuclear mRNA, suggesting that there was no additive effect for the co-depletion of both complexes. This result argues in favour of the hypothesis that SRSF3 and the RNA exosome complex are in the same pathway and suggests that SRSF3 could aid in the recruitment of the nuclear RNA exosome onto the target mRNAs.

**Figure 6:**
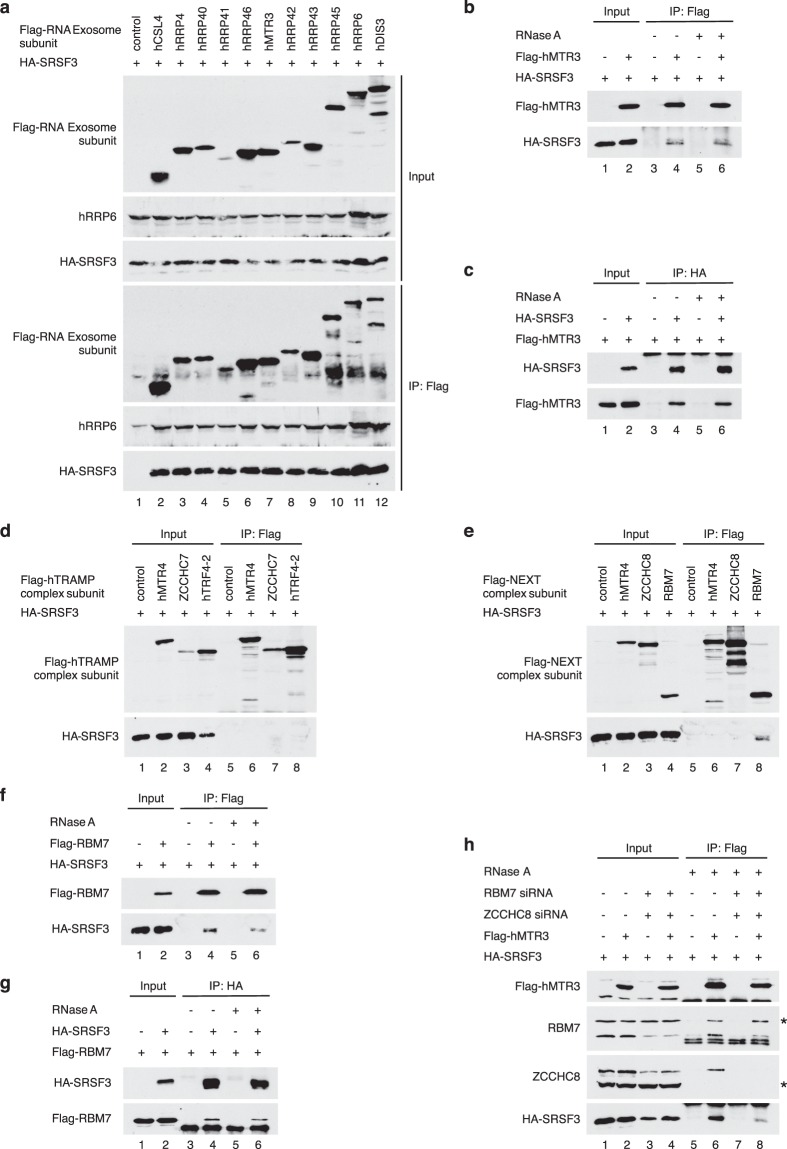
The RNA exosome and the NEXT complex interact with the splicing factor SRSF3. (**a**) HA-SRSF3 was co-expressed with individual Flag epitope-tagged RNA exosome subunits in HeLa cells as indicated. The top panels show western blotting of 1/10 of total extract (‘Input’), and the bottom panels show western blotting following immunoprecipitation with an anti-Flag antibody (IP: Flag). Anti-hRRP6 antibody was used as a positive control. (**b**) and (**c**) HA-SRSF3 and Flag-hMTR3 were expressed by transient transfection in HeLa cells as indicated. Cellular extracts were immunoprecipitated with an anti-Flag antibody (**b**) or an anti-HA antibody (**c**) in the presence or absence of RNase A. The immunoprecipitated complexes were then analyzed by western blotting using an anti-Flag polyclonal antibody (**c**) or an anti-HA antibody (**b**). (**d**) and (**e**) HA-SRSF3 was coexpressed with individual Flag epitope-tagged hTRAMP (**d**) or NEXT (**e**) complex subunits in HeLa cells. Cellular extracts were immunoprecipitated with an anti-Flag antibody and then the immunoprecipitated complexes were analyzed by western blotting using an anti-Flag polyclonal antibody to visualize the hTRAMP and NEXT complex subunits (top panel), or an anti-HA antibody to detect SRSF3 (bottom panel). (**f**) and (**g**) HA-SRSF3 and RBM7 Flag-tagged NEXT subunit were expressed by transient transfection in HeLa cells as indicated. Cellular extracts were immunoprecipitated with an anti-Flag antibody (**f**) or with an anti-HA antibody (**g**) in the presence or absence of RNase A. The immunoprecipitated complexes were then analyzed by western blotting using an anti-Flag polyclonal antibody (**g**) or an anti-HA antibody (**f**). (**h**) HA-SRSF3 was expressed by transient transfection in HeLa cells previously treated with control or specific siRNAs for the NEXT complex, either alone or together with the hMTR3 Flag-tagged RNA exosome subunit. Cellular extracts were immunoprecipitated with an anti-Flag antibody in the presence or absence of RNase A. The immunoprecipitated complexes were then analyzed by western blotting using an anti-Flag polyclonal antibody to visualize hMTR3 (top panel), anti-RBM7 or anti-ZCCHC8 antibodies (middle panels), or an anti-HA antibody to detect SRSF3 (bottom panel). * indicates non-specific bands.

To assay for a possible association between SRSF3 and the RNA exosome *in vivo*, each Flag-tagged subunit of the RNA exosome expressed in HeLa cells was immunoprecipitated, and co-purification of SRSF3 was assessed by western blotting. hRRP6, one of the ribonucleases associated with the core RNA exosome, was used as a positive control. These analyses revealed that SRSF3 was co-immunoprecipitated with each subunit of the RNA exosome (Fig. 6a), suggesting an association of SRSF3 with the RNA exosome. This interaction did not require the presence of RNA since SRSF3 still co-immunoprecipitated with the hMTR3 subunit of the RNA exosome following RNase treatment (Fig. 6b,c). In order to be certain that SRSF3 was not unspecifically immuno-precipitated, a control IP experiment using a non-specific IgG antibody was performed. The result, presented in Fig. S1, shows that neither the Flag-tagged transfected proteins nor SRSF3 were immuno-precipitated by the control antibody.

Taken together, our findings indicate that SRSF3 associates, either directly or indirectly, with the RNA exosome complex in cells. The RNA exosome consists of a catalytically inactive nine-subunit core that acquires its ribonucleolytic activity through its association with the two subunits, hRRP6 and hDIS3/hRRP44. Although active *in vitro*, the RNA exosome complex requires appropriate adaptors to be fully functional *in vivo*. Different subtypes of adaptors like the hTRAMP and NEXT complexes have been described in the nucleus of mammalian cells. These adaptors direct the RNA exosome to its target substrates, are specific for discrete processing/degradation pathways and can even recognize specific RNA features. We thus asked whether the RNA exosome could be recruited to SRSF3 by one of these adaptors. Interestingly, when Flag-tagged versions of each hTRAMP subunit were individually expressed along with HA-SRSF3 in HeLa cells, none of these hTRAMP components was able to pull down SRSF3 (Fig. 6d). However, the same experiment made with individual subunits of the NEXT complex showed that RBM7 co-precipitated with SRSF3 (Fig. 6e). This association was resistant to RNase treatment (Fig. 6f) and was also found in the reverse experiment in which HA-SRSF3 was immunoprecipitated (Fig. 6g). Taken together, these results show that SRSF3 interacts with both the RNA exosome and the NEXT complex and suggest that the nuclear RNA exosome can be recruited onto specific target mRNAs by SRSF3 via its association with the NEXT complex. In order to confirm this hypothesis, the NEXT subunits, RBM7 and ZCCHC8, were depleted from cells using specific siRNAs, and the exosome subunit hMTR3 immunoprecipitated to determine whether SRSF3 was still associated with the RNA exosome (Fig. 6h). As expected, immunoprecipitation of the RNA exosome complex (through its hMTR3 subunit) allowed co-immunoprecipitation of SRSF3 together with the NEXT components, RBM7 and ZCCHC8. However, when expression of RBM7 and ZCCHC8 was downregulated, co-immunoprecipitation of SRSF3 with the RNA exosome complex (again through its hMTR3 subunit) was greatly impaired. It has to be noted that downregulation of the NEXT subunits (RBM7 and ZCCHC8) induces a decrease in the level of SRSF3, further suggesting that NEXT and the SRSF3 protein are linked. Taken together, these results argue strongly in favor of a direct interaction between SRSF3 and the NEXT subunit RBM7 and suggest that the interaction between SRSF3 and the RNA exosome is mediated by RBM7.

### Functional implications of the SRSF3 and RNA exosome interaction

In order to study the functional relevance of the interactions we had found between the SR protein SRSF3 and the RNA exosome, we then assessed the effect of depletion of various components of these complexes on the steady-state levels of our reporter mRNAs. For this purpose, HeLa cells were treated with siRNAs targeting either (i) the RNA exosome core subunit hRRP40, (ii) the SRSF3 protein, (iii) the NEXT complex subunits RBM7 and ZCCHC8, or (iv) the RNA helicase hMTR4.

**Figure 7:**
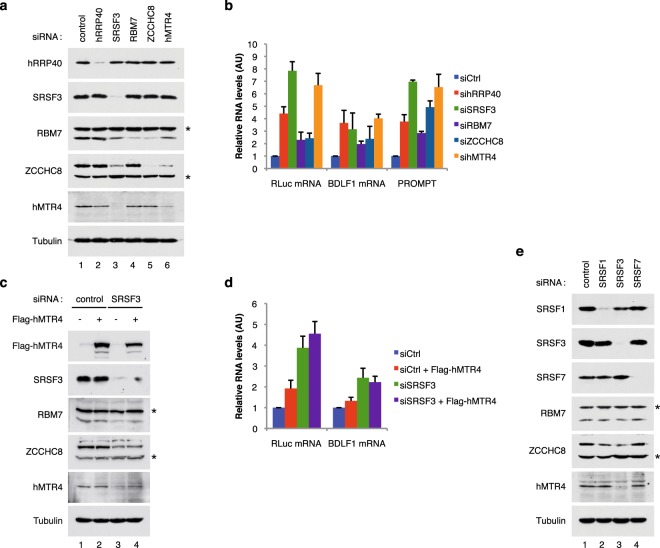
The SRSF3 protein and the NEXT complex associated with the RNA exosome are functionally connected to degrade intronless mRNAs. (**a**) Western blot analysis showing protein depletion upon the indicated siRNA treatments. Anti-Tubulin antibody was used as a loading control. * Indicates non-specific bands. (**b**) *mRNA stabilization by exosome depletion:* RT-qPCR analysis of nuclear RNAs from HeLa cells transfected with the indicated siRNAs together with the Renilla luciferase and BDLF1 intronless constructs. proEXT1 PROMPT served as a positive control. Data are displayed as mean values normalized to the control siRNA and GAPDH mRNA as an internal control. Error bars represent the s.d. from three independent experiments (*n* = 3). (**c**) Western blotting analysis showing the overexpression of the Flag tagged-hMTR4 protein and depletion of the SRSF3 protein after siRNA treatment. Anti-Tubulin antibody was used as a loading control. (**d**) RT-qPCR analysis of nuclear RNAs from HeLa cells transfected with the indicated siRNAs together with the Renilla luciferase and BDLF1 intronless constructs. Data are displayed as mean values normalized to the control siRNA and GAPDH mRNA as an internal control. Error bars represent the s.d. from three independent experiments (*n* = 3). (**e**) Western blotting analysis showing expression of the NEXT components RBM7, ZCCHC8 and hMTR4 in HeLa cells transfected with either a control siRNA (lane 1), or a siRNA specific for SRSF1 (lane 2), SRSF3 (lane 3) or SRSF7 (lane 4). Anti-Tubulin antibody was used as a loading control. *Indicates non-specific bands.

Knockdown efficiencies were controlled by western blotting analyses (Fig. 7a) and by quantification of a specific ncRNA known to be degraded by the RNA exosome and the NEXT complex, the PROMPT proEXT1 (Fig. 7b)^27^. As shown previously, RT-qPCR analyses revealed that the level of RLuc and BDLF1 mRNAs increased upon depletion of hRRP40 and SRSF3 (Fig. 7b). Depletion of hMTR4 also led to an increase in mRNA accumulation similar to that observed with the depletion of SRSF3 or the RNA exosome. RBM7 or ZCCHC8 down-regulation also led to an increased accumulation of the reporter mRNA, but their impact was not as efficient as the down-regulation of hMTR4 or SRSF3. It is important to note that the downregulation of RBM7 and ZCCHC8 was not as efficient as the downregulation of SRSF3. However, down-regulation of hMTR4 and SRSF3 also decreased the expression level of both RBM7 and ZCCHC8 (Fig. 7a), which might explain why the depletion of these two factors had a stronger impact. In summary, the data collectively suggest that SRSF3, the NEXT complex and the RNA exosome are linked in the process of nuclear mRNA decay.

The observation that SRSF3’s depletion impacts on RBM7, ZCCHC8 and hMTR4 levels (Fig. 7a) suggests that a strong link exists between the NEXT complex and the SR protein, SRSF3. In order to demonstrate that these results were not due to an off-target effect of the specific siRNA used, we repeated the experiments with two other independent siRNAs targeting SRSF3 and obtained similar results (Fig. S2). In order to prove that the effect seen at the protein level was not the result of an effect of SRSF3 on hMTR4 stability or splicing, both the amount and the integrity of the hMTR4 mRNA were controlled by RT-PCR in cells treated with the siRNA directed against SRSF3. From this analysis we concluded that downregulation of SRSF3 does not significatively affect the expression level or splicing of hMTR4 mRNA (Fig. S2). This argues in favor of a direct impact of SRSF3 on the stability of the hMTR4 protein. This observation could suggest that SRSF3 exerts its effect on the stability of intronless mRNAs via a downregulation of hMTR4 expression. To test this hypothesis, we analyzed, by RT-qPCR, the stability of the BDLF1 and Luciferase reporter genes in cells overexpressing hMTR4 - and in which SRSF3 was first downregulated (Fig. 7d). As previously found, downregulation of SRSF3 impacted hMTR4, RBM7 and ZCCHC8 expression levels. However, in these conditions, overexpression of hMTR4 did not rescue the expression level of RBM7 and ZCCHC8 (Fig. 7c). Moreover, the RLuc and BDLF1 mRNAs accumulated in the cell nucleus at similar levels as in the absence of hMTR4 overexpression (Fig. 7d). This demonstrates that SRSF3’s effect on the stability of the intronless mRNAs is not due to hMTR4 downregulation alone.

Finally, in order to determine whether the link between the SR protein SRSF3 and the RNA exosome is specific for SRSF3, the expression of two other SR proteins, SRSF1 (ASF/SF2) and SRSF7 (9G8), was down-regulated and the level of the NEXT complex components assessed by western blot (Fig. 7e). SRSF3 was the only protein of the three whose down-regulation was able to disturb the expression of NEXT complex components. This suggests that SRSF3 makes a specific contribution to mRNA decay that other SR proteins do not.

## Discussion

In this study, we demonstrate that some mRNAs synthesized from intronless genes - which is the case for most herpesviruses mRNAs produced during the viral productive cycle - are unstable and are degraded in the nucleus. We also show that the splicing factor SRSF3 is involved in the nuclear destabilization of these intronless mRNAs by the RNA exosome. The RNA exosome complex is recruited onto the target mRNAs by the NEXT complex associated with SRSF3 via its RBM7 component. In addition, SRSF3 appears to play an important role in the stabilization of the NEXT complex. The interaction between the NEXT complex, the RNA exosome and the SRSF3 protein suggests that the mRNA degradation machinery is co-transcriptionally recruited onto the mRNA.

Herpesvirus mRNAs synthesized from intronless genes, inefficiently accumulate in the cytoplasm in the absence of a specific viral protein^40,53,54^. In the case of EBV, viral mRNAs synthesized from intronless genes, not only accumulate in the cytoplasm but are also stabilized in the cell nucleus. However, it is surprising to observe that the BDLF1 mRNA accumulates in the cytoplasm, although its nuclear half-life is over 7 hours. In effect, it should not be expected to stay in the nucleus for such a long period if it is efficiently exported by EB2. This suggests that EB2 has only a minor role in mRNA export and that its major function is to stabilize its target mRNAs. It has been shown that some RNA molecules can spend hours in the nucleus before being exported to the cytoplasm. mRNA nuclear retention can effectively buffer the burst of mRNA transcription which could induce temporal fluctuations in the level of cytoplasmic mRNA^55^. Since, in our assay, the BDLF1 gene is under the control of a very strong promoter, we can hypothesize that the high level of BDLF1 mRNA produced may be retained in the nucleus and protected from nuclear degradation when EB2 is expressed.

**Figure 8:**
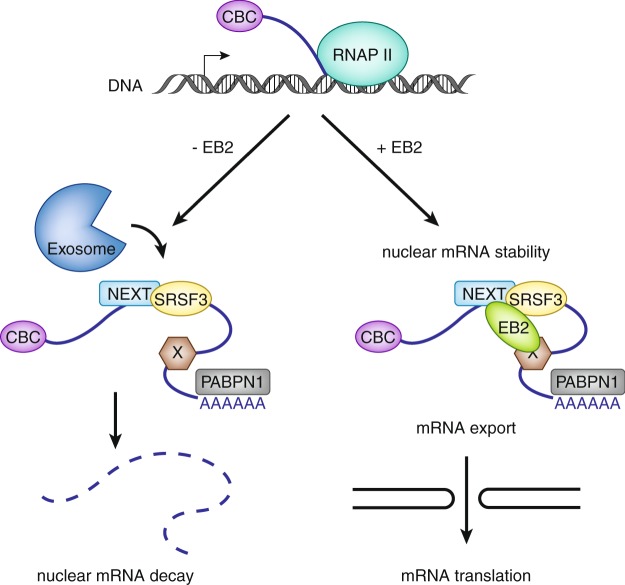
Model. During their transcription, intronless nascent mRNAs associate with numerous RNA-binding proteins like the cap-binding complex (CBC), the nuclear poly(A)-binding protein (PABPN1), and some spliceosome components such as the SRSF3 protein. In the absence of EB2, the association of the splicing factor SRSF3 to newly synthesized mRNAs led to the recruitment of the RNA exosome via the NEXT complex, and consequently to their degradation in the nucleus. In the presence of EB2, EB2 stabilizes these intronless mRNAs in the nucleus and then allow their export to, and translation, in the cytoplasm.

Based on our findings, we propose a novel model for the coupling between pre-mRNA splicing and nuclear RNA quality control revealed by our study on the function of the EBV protein EB2 (Fig. 8). In the absence of EB2 expression, the SR protein SRSF3 loaded onto the viral mRNAs expressed from intronless genes, recruits the splicing machinery and the NEXT complex together with the nuclear RNA exosome, leading to the destabilization of the mRNAs and their decay. By contrast, when EB2 is expressed, it interacts with SRSF3^46,47^, hence preventing the nuclear RNA exosome’s access to the mRNAs that are then protected from the degradation machinery. However, overexpression of EB2 does not appear to inhibit the interaction between SRSF3 and the NEXT complex suggesting that EB2 and RBM7 do not interact with the same domain of SRSF3. Another way in which EB2 could interfere with RNA exosome recruitment, could be by changing the ability of the SRSF3/NEXT complex loaded onto the mRNA, to interact with the RNA exosome. Concomitantly, the interaction between EB2 and the TAP-NXF1/p15 complex favors mRNAs export from the nucleus^42^ and their translation in the cytoplasm^36,52^.

Interestingly, our present work demonstrating the implication of specific components of the spliceosome (i. e. SRSF3) and the RNA exosome in the nuclear degradation of intronless RNAs extends a previous work from Bresson *et al*. showing that the nuclear poly(A)-binding protein and the exosome are also implicated in the nuclear degradation of intronless RNAs^56^.

As discussed above, our work provides evidence of an interaction between SRSF3 and the NEXT complex. In this context, it is noteworthy that depletion of SRSF3 induces a concomitant depletion of all three components of the NEXT complex - RBM7, ZCCHC8 and hMTR4. Similarly, depletion of MTR4 correlates with a depletion of both RBM7 and ZCCHC8. Taken together, these observations suggest that SRSF3 contributes to the stabilization of the NEXT complex, hence also increasing the stability of its components. However, from our results, it would appear that the depletion of hMTR4 is complete when SRSF3 is downregulated. This is surprising since only a fraction of the hMTR4 protein pool is expected to be associated with the NEXT complex. In effect, in human cells, the hMTR4 RNA helicase which is required for all activities of the nuclear RNA exosome^22^ is associated with the NEXT, PAXT and hTRAMP complexes. However, the antibody used in our assays to detect hMTR4 is not very efficient and only detects hMTR4 expressed at a high level. The level of remaining hMTR4, following SRSF3 depletion, is probably insufficient to be detected by the antibody. Nevertheless, the modification of the protein level of the different components of the NEXT complex observed when SRSF3 is depleted, argues in favor of the formation of a stable complex between SRSF3 and NEXT and comforts the hypothesis that SRSF3 plays an important role in RNA exosome activity. In addition, the recently published results of independent proteomics analysis^21,22,27^ demonstrating that RBM7, ZCCHC8, hMTR4 and SRSF3 co-purify, completely support our conclusion that SRSF3 interacts with the NEXT complex and not only with RBM7, outside of the NEXT context.

In the cell nucleus, several types of coding and non-coding RNAs are synthesized and processed. To ensure the survival of cells, only functional competent RNAs must be produced. All the aberrant and inproperly processed transcripts have to be recognized and degraded by RNA quality control mechanisms^57,58^. mRNA expression is the result of a coupling between transcription by RNA polymerase II and pre-mRNA processing reactions such as 5′-end capping, splicing, 3′-end maturation and mRNP formation. All these events can affect the localization, translation and degradation of the mRNA. Recent data have shown that the 5′cap of the mRNA associated with the cap-binding complex (CBC) is a type of hub that mediates key events in the life of the mRNA^59,60^. Indeed, as recently demonstrated, the CBC is very important both for the recruitment of the mRNA export machinery and the recruitment of the RNA exosome through the NEXT complex^27,61^, suggesting a direct link between mRNA 5′-end capping and nuclear RNA surveillance. Interestingly, it has also been shown that the CBC is necessary, although not sufficient, for co-transcriptional spliceosome assembly in yeast^62^. This demonstration of an essential link between the CBC and spliceosome assembly indicates that 5′-end capping couples transcription to pre-mRNA splicing and probably also to mRNA decay via recruitment of the RNA exosome by specific splicing factors. Our results support a direct coupling between the splicing and the mRNA degradation machineries. Thus, in addition to the recruitment of NEXT by the CBC complex, there appears to be an alternative mode of recruitment of the nuclear RNA exosome involving specific spliceosome components.

## Materials and Methods

### Plasmids

pCMV-Renilla Luciferase, pCMV-HA-SRSF3, pCMV-EB1, pCI-Flag-EB2 and pCMV-BDLF1 have been described elsewhere^34,36,40,47^. For pTRE2-BDLF1, the EBV BDLF1 CDS was amplified by PCR and cloned into the BamHI and XbaI sites of the pTRE2 expression plasmid (Clontech). The pTet-On vector was supplied by Clontech (Tet-Off and Tet-On gene expression systems). Individual Flag-tagged RNA exosome components, hTRAMP and NEXT subunits were a generous gift from Drs Frederick W. Alt and Torben Heick Jensen^21,63^. All plasmid sequences are available upon request.

### Cell culture and transfections

HeLa cells were grown at 37 °C in DMEM supplemented with 10% Foetal Bovine Serum (FBS) and penicillin-streptomycin. HEK293_EBV∆BMLF1_ cells^40^ were maintained in DMEM supplemented with 10% FBS, penicillin-streptomycin and hygromycin B (100 µg/ml). Raji cells were maintained in RPMI supplemented with 10% FBS and penicillin-streptomycin. 48 h prior to DNA transfection, siRNAs were transfected using INTERFERin transfecting reagent (Polyplus-transfection) at 20 nM final concentration, following the manufacturer’s instructions (see Table S1 of Supplementary Data for the list of siRNAs used). Plasmid transfections were performed using cationic polymers (jetPEI from Polyplus-transfection) as specified by the manufacturer and 24 h or 48 h after DNA transfection, cells were collected for further analysis.

### Virus production and titration

HEK293_EBV∆BMLF1_ cells were transfected with pCMV-EB1 in order to activate the viral productive cycle. Cell supernatants were harvested 48 h post-transfection and filtered through a 0.45 -µm pore size filter. Raji cells (1 × 10^5^) were incubated with 1 ml of cell supernatant for 6–8 h at 37 °C in a 24-well plate. Cells were then washed, re-suspended in 1 ml of RPMI supplemented with 10% FBS and penicillin-streptomycin and incubated for an additional 72 h at 37 °C. GFP-expressing Raji cells were quantified by FACS analysis.

### Pulse-chase mRNA decay assays

HeLa cells were transfected in 60 mm dishes with the pTet-On vector and the pTRE2-BDLF1 plasmid. 24 h after transfection, 1 μg/ml of doxycycline (Sigma-Aldrich) was added to repress transcription. Cells were then harvested at the time points indicated in individual experiments. Actinomycin D treatment of HeLa cells was performed using 5 μg/ml of actinomycin D (Sigma-Aldrich).

### RNA extraction and RT-qPCR analysis

Nuclear and cytoplasmic RNAs extraction were performed as previously described^36^ and reverse-transcribed using the qScript cDNA Synthesis Kit (Quanta BioSciences). Standard PCRs were performed using GoTaq DNA polymerase (Promega) and the PCR-amplified fragments analyzed on 2% agarose gels. All PCR reactions were made using several dilutions of the RT products in order to insure that the RT-PCR results were in the linear range of the PCR reaction. We used ß-actin mRNA and U6 snRNA as internal controls. qPCRs were performed using FastStart Universal SYBR Green Master (Rox) (Roche) on an Applied Biosystems 7000 thermocycler. Cycling conditions were 5 min at 95 °C and 45 cycles of 15 s at 95 °C, 30 s at 60 °C on a 96-well thermoblock. This program was followed by melting curve analysis in order to verify the specificity of the PCR products. PCR results were normalized with the parallel amplification of GAPDH mRNA. The various set of primers used in the study are listed in Table S2 of Supplementary Data.

### Co-immunoprecipitation assays

48 h after transfection, cells from a 100 mm plate were washed in PBS (Life Technologies) and collected in 0.5 ml of RSB100 buffer (10 mM Tris-HCl pH 7.4, 100 mM NaCl, 2.5 mM MgCl_2_), containing 0.5% Triton X-100 and protease inhibitors (Roche). Cells were gently lysed by sonication and centrifuged (4000 g, 15 min) at 4 °C. Supernatants were incubated with 2 μg of a mouse anti‐Flag M2 antibody (Sigma-Aldrich, F3165) or 2 μg of a mouse anti‐HA antibody (Sigma-Aldrich, H3663) overnight at 4 °C, 10 r.p.m. rotation. Protein-antibody complexes were recovered with 40 μl of Protein G Sepharose 4 Fast Flow beads (GE Healthcare Life Sciences) during 2 h at 4 °C, 10 r.p.m. and recovered complexes were washed extensively with RSB100/0.5% Triton X-100 buffer. RNase treatment was performed by adding 2 μl of RNase A (10 mg/ml) to the incubation mixture where indicated. Immunopurified proteins were then resuspended in SDS‐loading buffer and analysed by western blotting.

### RNA immunoprecipitation

RIPs were carried out from uncrosslinked extracts. Briefly, 10^8^ cells were washed in PBS and then resuspended in 1 ml of RSB100 buffer (10 mM Tris-HCl pH 7.4, 100 mM NaCl, 2.5 mM MgCl_2_), containing 0.5% Triton X-100, protease inhibitors (Roche) and RNasin (Promega). Extracts obtained after centrifugation were incubated with 2 μg of a mouse anti‐HA antibody (Sigma-Aldrich, H3663) overnight at 4 °C. PureProteome ProteinA/G Mix Magnetic Beads (Merck Millipore) were mixed and coated with PBS + 10% BSA, supplemented with tRNA and RNasin overnight. After re-equilibration with RSB100 buffer, the beads were added to the lysate for 2 h at 4 °C before extensive washing in RSB100 buffer containing 0.5% Triton X-100 and 0.05% NP-40. Half of the immunoprecipitates were analyzed by Western blotting. The other half were treated 15 min at 55 °C with proteinase K (0.5 mg/ml) and SDS (0.1%), and the RNAs were recovered by phenol/chloroforme extraction and ethanol precipitation.

### Western blot analysis

Cell lysates were prepared in RSB100/0.5% Triton X-100 and protease inhibitors (Roche) buffer and quantified by Bradford assay. Equivalent amounts of each sample were resolved by SDS-PAGE and analysed by western blotting using the following antibodies: mouse anti-Tubulin (Sigma-Aldrich, clone B-5-1-2; 1:1000), mouse monoclonal anti-EB1 (Z125 antibody; 1:1000)^64^, rabbit polyclonal anti-EB2 (1:500)^40^, mouse monoclonal anti-gp350 (1:500)^65^, rabbit polyclonal anti-Flag (Sigma-Aldrich, F7425; 1:2500), rabbit polyclonal anti-HA (Sigma-Aldrich, H6908; 1:1000), mouse monoclonal anti-SRSF1 (Life Technologies, clone 7B4; 1:1000), mouse monoclonal anti-SRSF3 (Life Technologies, clone 96; 1:1000), rabbit polyclonal anti-SRSF7 (Sigma-Aldrich, HPA043850; 1:1000), rabbit polyclonal anti-XRN2 (a gift from N J Proudfoot; 1:1000), rabbit polyclonal anti-DCP2 (a gift from M Kiledjian; 1:1000), mouse polyclonal anti-hDIS3 (Abnova, H00022894-A01; 1:1000), rabbit polyclonal anti-hRRP40, anti-hRRP46 and anti-hRRP6 (a gift from Ger J M Pruijn; 1:1000), human anti-hMTR4 (a gift from Ger J M Pruijn; 1:1000), rabbit polyclonal anti-ZCCHC8 (Sigma-Aldrich, HPA037484; 1:1000) or rabbit polyclonal anti-RBM7 (Sigma-Aldrich, HPA013993; 1:1000).

### Statistical analysis

All results are expressed as means ± standard deviations of experiments independently repeated at least three times. Unpaired Student’s *t*-test was used to evaluate the statistical difference between samples and significance was evaluated with *P*-values as follows: * *P* < 0.05; ** *P* < 0.01; *** *P* < 0.001.

## Electronic supplementary material

Supplementary Information

## References

[1] Müller-McNicoll M, Neugebauer KM (2013). How cells get the message: dynamic assembly and function of mRNA-protein complexes. Nat. Rev. Genet..

[2] Fasken MB, Corbett AH (2005). Process or perish: quality control in mRNA biogenesis. Nat. Struct. Mol. Biol..

[3] Singh G, Pratt G, Yeo GW, Moore MJ (2015). The Clothes Make the mRNA: Past and Present Trends in mRNP Fashion. Annu. Rev. Biochem..

[4] Cho E-J, Takagi T, Moore CR, Buratowski S (1997). mRNA capping enzyme is recruited to the transcription complex by phosphorylation of the RNA polymerase II carboxy-terminal domain. Genes Dev..

[5] David CJ, Manley JL (2011). The RNA polymerase C-terminal domain. Transcription.

[6] Pickrell JK, Pai AA, Gilad Y, Pritchard JK (2010). Noisy Splicing Drives mRNA Isoform Diversity in Human Cells. PLoS Genet..

[7] Eberle AB, Visa N (2014). Quality control of mRNP biogenesis: Networking at the transcription site. Semin. Cell Dev. Biol..

[8] Davidson L, Kerr A, West S (2012). Co-transcriptional degradation of aberrant pre-mRNA by Xrn2. EMBO J..

[9] Liu Q, Greimann JC, Lima CD (2006). Reconstitution, activities, and structure of the eukaryotic RNA exosome. Cell..

[10] Lebreton A, Tomecki R, Dziembowski A, Séraphin B (2008). Endonucleolytic RNA cleavage by a eukaryotic exosome. Nature..

[11] Schmid M, Jensen TH (2008). Quality control of mRNP in the nucleus. Chromosoma..

[12] Lykke-Andersen S, Brodersen DE, Jensen TH (2009). Origins and activities of the eukaryotic exosome. J. Cell Sci..

[13] LaCava J (2005). RNA degradation by the exosome is promoted by a nuclear polyadenylation complex. Cell..

[14] Wyers F (2005). Cryptic Pol II Transcripts Are Degraded by a Nuclear Quality Control Pathway Involving a New Poly(A) Polymerase. Cell..

[15] Vaňáčová Š (2005). A New Yeast Poly(A) Polymerase Complex Involved in RNA Quality Control. PLoS Biol..

[16] Butler JS, Mitchell P (2010). Rrp6, Rrp47 and cofactors of the nuclear exosome. Adv. Exp. Med. Biol..

[17] Mitchell P (2003). Rrp47p is an exosome-associated protein required for the 3′ processing of stable RNAs. Mol. Cell. Biol..

[18] Lemay J-F, Lemieux C, St-André O, Bachand F (2010). Crossing the borders: poly(A)-binding proteins working on both sides of the fence. RNA Biol..

[19] Lemay J-F (2010). The Nuclear Poly(A)-Binding Protein Interacts with the Exosome to Promote Synthesis of Noncoding Small Nucleolar RNAs. Mol. Cell..

[20] Fasken MB (2011). Air1 Zinc Knuckles 4 and 5 and a Conserved IWRXY Motif Are Critical for the Function and Integrity of the Trf4/5-Air1/2-Mtr4 Polyadenylation (TRAMP) RNA Quality Control Complex. J. Biol. Chem..

[21] Lubas M (2011). Interaction Profiling Identifies the Human Nuclear Exosome Targeting Complex. Mol. Cell..

[22] Meola N (2016). Identification of a Nuclear Exosome Decay Pathway for Processed Transcripts. Mol. Cell..

[23] Sloan KE, Schneider C, Watkins NJ (2012). Comparison of the yeast and human nuclear exosome complexes. Biochem. Soc. Trans..

[24] Wolin SL, Sim S, Chen X (2012). Nuclear noncoding RNA surveillance: is the end in sight?. Trends Genet..

[25] Falk S (2016). Structure of the RBM7-ZCCHC8 core of the NEXT complex reveals connections to splicing factors. Nat. Commun..

[26] Guo TB (2003). Spermatogenetic Expression of RNA-Binding Motif Protein 7, a Protein That Interacts With Splicing Factors. J. Androl..

[27] Andersen PR (2013). The human cap-binding complex is functionally connected to the nuclear RNA exosome. Nat. Struct. Mol. Biol..

[28] Nag A, Steitz JA (2012). Tri-snRNP-associated proteins interact with subunits of the TRAMP and nuclear exosome complexes, linking RNA decay and pre-mRNA splicing. RNA Biol..

[29] Rickinson, A. B. & Kieff, E. Epstein-Barr virus. in *Fields’ Virology* (eds. Knipe, D. M. et al.) **2**, 2655–2700 (Lippincott - Williams & Wilkins, 2007).

[30] Babcock GJ, Decker LL, Volk M, Thorley-Lawson DA (1998). EBV persistence in memory B cells *in vivo*. Immunity..

[31] Laichalk LL, Thorley-Lawson DA (2005). Terminal differentiation into plasma cells initiates the replicative cycle of Epstein-Barr virus *in vivo*. J Virol..

[32] Luo M, Reed R (1999). Splicing is required for rapid and efficient mRNA export in metazoans. Proc Natl Acad Sci..

[33] Le Hir H, Gatfield D, Izaurralde E, Moore MJ (2001). The exon-exon junction complex provides a binding platform for factors involved in mRNA export and nonsense-mediated mRNA decay. Embo J..

[34] Batisse J, Manet E, Middeldorp J, Sergeant A, Gruffat H (2005). Epstein-Barr virus mRNA export factor EB2 is essential for intranuclear capsid assembly and production of gp350. J. Virol..

[35] Buisson M (1989). The Epstein-Barr virus (EBV) early protein EB2 is a posttranscriptional activator expressed under the control of EBV transcription factors EB1 and R. J. Virol..

[36] Ricci EP (2009). Translation of intronless RNAs is strongly stimulated by the Epstein-Barr virus mRNA export factor EB2. Nucleic Acids Res..

[37] Boyer JL, Swaminathan S, Silverstein SJ (2002). The Epstein-Barr virus SM protein is functionally similar to ICP27 from herpes simplex virus in viral infections. J Virol..

[38] Swaminathan S (2005). Post-transcriptional gene regulation by gamma herpesviruses. J Cell Biochem..

[39] Hiriart E (2003). A region of the Epstein-Barr virus (EBV) mRNA export factor EB2 containing an arginine-rich motif mediates direct binding to RNA. J. Biol. Chem..

[40] Gruffat H (2002). Epstein-Barr virus mRNA export factor EB2 is essential for production of infectious virus. J. Virol..

[41] Hiriart E (2003). A novel nuclear export signal and a REF interaction domain both promote mRNA export by the Epstein-Barr virus EB2 protein. J. Biol. Chem..

[42] Juillard F (2009). Epstein-Barr virus protein EB2 contains an N-terminal transferable nuclear export signal that promotes nucleocytoplasmic export by directly binding TAP/NXF1. J Virol..

[43] Katahira J (2012). mRNA export and the TREX complex. Biochim. Biophys. Acta..

[44] Hiriart E (2005). Interaction of the Epstein-Barr virus mRNA export factor EB2 with human Spen proteins SHARP, OTT1, and a novel member of the family, OTT3, links Spen proteins with splicing regulation and mRNA export. J. Biol. Chem..

[45] Verma D, Swaminathan S (2008). Epstein-Barr virus SM protein functions as an alternative splicing factor. J Virol..

[46] Verma D, Bais S, Gaillard M, Swaminathan S (2011). Epstein-Barr Virus SM protein utilizes cellular splicing factor SRp20 to mediate alternative splicing. J Virol..

[47] Juillard F (2012). Epstein-Barr virus protein EB2 stimulates cytoplasmic mRNA accumulation by counteracting the deleterious effects of SRp20 on viral mRNAs. Nucleic Acids Res..

[48] Huang Y, Steitz JA (2001). Splicing factors SRp20 and 9G8 promote the nucleocytoplasmic export of mRNA. Mol Cell..

[49] Nicewonger J, Suck G, Bloch D, Swaminathan S (2004). Epstein-Barr Virus (EBV) SM Protein Induces and Recruits Cellular Sp110b To Stabilize mRNAs and Enhance EBV Lytic Gene Expression. J. Virol..

[50] Sahin BB, Patel D, Conrad NK (2010). Kaposi’s Sarcoma-Associated Herpesvirus ORF57 Protein Binds and Protects a Nuclear Noncoding RNA from Cellular RNA DecayPathways. PLoS Pathogen..

[51] Garneau NL, Wilusz J, Wilusz CJ (2007). The highways and byways of mRNA decay. Nat. Rev. Mol. Cell Biol..

[52] Mure F (2018). Epstein-Barr virus protein EB2 stimulates translation initiation of mRNAs through direct interactions with both PABP and eIF4G. J. Virol..

[53] Han Z, Swaminathan S (2006). Kaposi’s sarcoma-associated herpesvirus lytic gene ORF57 is essential for infectious virion production. J Virol..

[54] Sandri-Goldin RM (2011). The many roles of the highly interactive HSV protein ICP27, a key regulator of infection. Future Microbiol..

[55] Bahar Halpern K (2015). Nuclear Retention of mRNA in Mammalian Tissues. Cell Rep..

[56] Bresson SM, Conrad NK (2013). The Human Nuclear Poly(A)-Binding Protein Promotes RNA Hyperadenylation and Decay. PLOS Genet..

[57] Libri D (2002). Interactions between mRNA Export Commitment, 3′-End Quality Control, and Nuclear Degradation. Mol. Cell. Biol..

[58] Bousquet-Antonelli C, Presutti C, Tollervey D (2000). Identification of a Regulated Pathway for Nuclear Pre-mRNA Turnover. Cell..

[59] Giacometti S (2017). Mutually Exclusive CBC-Containing Complexes Contribute to RNA Fate. Cell Rep..

[60] Fan J (2017). Exosome cofactor hMTR4 competes with export adaptor ALYREF to ensure balanced nuclear RNA pools for degradation and export. EMBO J..

[61] Hallais M (2013). CBC-ARS2 stimulates 3′-end maturation of multiple RNA families and favors cap-proximal processing. Nat. Struct. Mol. Biol..

[62] Görnemann J, Kotovic KM, Hujer K, Neugebauer KM (2005). Cotranscriptional Spliceosome Assembly Occurs in a Stepwise Fashion and Requires the Cap Binding Complex. Mol. Cell..

[63] Basu U (2011). The RNA exosome targets the AID cytidine deaminase to both strands of transcribed duplex DNA substrates. Cell..

[64] Gruffat H, Manet E (2002). & Sergeant, A. MEF2-mediated recruitment of class II HDAC at the EBV immediate early gene BZLF1 links latency and chromatin remodeling. EMBO Rep..

[65] Hessing M, van Schijndel HB, van Grunsven WM, Wolf H, Middeldorp JM (1992). Purification and quantification of recombinant Epstein-Barr viral glycoproteins gp350/220 from Chinese hamster ovary cells. J Chromatogr..

