# Supplementary material for: The splicing factor SRSF3 is functionally connected to the nuclear RNA exosome for intronless mRNA decay
Source: Sci Rep. 2018 Aug 27;8:12901. doi: 10.1038/s41598-018-31078-1 (PMC6110769; doi:10.1038/s41598-018-31078-1)
Supplement: Supplementary file 1 — Supplementary Information [file 41598_2018_31078_MOESM1_ESM.pdf]

The splicing factor SRSF3 is functionally connected to the nuclear RNA exosome for intronless mRNA decay

Fabrice Mure[1,2,3,4,5], Antoine Corbin[1,2,3,4,5], Nour El Houda Benbahouche[6], Edouard Bertrand[6], Evelyne Manet[1,2,3,4,5] and Henri Gruffat[1,2,3,4,5 #].

Supplementary Information

Supplementary Table S1. siRNAs

RNAi target	sense strand	anti-sense strand
Firefly (control)	GCGGAAUACUUCGAAAUGUdTdT	ACAUUUCGAAGUAUUCCGCdTdT
SRSF1	CCAAGGACAUUGAGGACGUdTdT	ACGUCCUCAAUGUCCUUGGdTdT
SRSF3	GGAAAUAGAAGACAGUUUGdTdT	CAAACUGUCUUGUAUUUCCdTdT
SRSF3#2	GAGUGGAACUGUCGAAUGGdTdT	CCAUUCGACAGUUCCACUCdTdT
SRSF3#3	CGAAGUGUGUGGGUUGCUAdTdT	UAGCAACCCACACACUUCGdTdT
SRSF7	AGGAGAGUUAGAAAGGGCUdTdT	AGCCCUUUCUAACUCUCCUdTdT
XRN2	GAGUACAGAUGAUCAUGUUdTdT	AACAUGAUCAUCUGUACUCdTdT
DCP2	GGACUGGCUUUCUCGAAGAdTdT	UCUUCGAGAAAGCCAGUCCdTdT
hRRP40 (EXOSC3)	CACGCACAGUACUAGGUCAdTdT	UGACCUAGUACUGUGCGUGdTdT
hRRP46 (EXOSC5)	CAACAAGGCCACACUCGAAdTdT	UUCGAGUGUGGCCUUGUUGdTdT
hRRP6 (EXOSC10)	CCAGUUAUACAGACCUAUAdTdT	UAUAGGUCUGUAUAACUGGdTdT
hDIS3/hRRP44	AGGUAGAGUUGUAGGAAUAdTdT	UAUUCCUACAACUCUACCUdTdT
hMTR4 (SKIV2L2)	CAAUUAAGGCUCUGAGUAAdTdT	UUACUCAGAGCCUUAAUUGdTdT
ZCCHC8	GGAAUGUACCUCAGGAUAAdTdT	UUAUCCUGAGGUACAUUCCdTdT
RBM7	GGAUAAAGGCAUUGCUUAAdTdT	UUAAGCAAUGCCUUUAUCCdTdT

Supplementary Table S2. PCR and qPCR primers

Target	Forward	Reverse
U6 snRNA	CGCTTCGGCAGCACATATAC	AAAATATGGAACGCTTCACGA
ß-actin	GCTGCGTGTGGCTCCCGAGGAG	ATCTTCATTGTGCTGGGTGCCAG
BMRF1	CCAGACATACGGTCAGTCCATCTC	TGCTTCACTTTCTTGGGGTGC
BDLF1	CAGATTTGAAAGTGGTAGTGTC	TTATCTTAACCAGCAAGTGGCCG
BdRF1	CACTATCAGGTAACGCAGGAG	TCAAGCCAGCGTTTATTCAGC
BFRF3	GGGAGGCTCAAAGAAGTTACCTG	ATGAAGAAACAGAGGGGGTCGC
hMTR4 (P1 + P2)	GGAGATGAGCTGTTCAGCGT	TTTCCGCCCTTTCTGGTCTC
hMTR4 (P3 + P4)	GCAGGTGATTTGGCCAAAGG	TCTCTTTGCTACAGCGCAGA
hMTR4 (P5 + P6)	GCCTAACTCTGGTGAACTGGA	TACAAGTAGAGGCTGGCAGC
SRSF3	ATGCATCGTGATTCCTGTCC	CATTTGACCTAGATCGACTACG
GAPDH_qPCR	AGCCACATCGCTCAGACAC	GCCCAATACGACCAAATCC
Renilla_qPCR	TGGAGAATAACTTCTTCGTGAAAAC	GCTGCAAATTCTTCTGGTTCTAA
BMRF1_qPCR	AGGAGTGCTGCAGGTAAACC	GCTCTGGTGATTCTGCCACT
BDLF1_qPCR	CGCAGACATGCTCGATGTA	GTAGTGGTGCCCCAGGTATG
BdRF1_qPCR	CGGAGTGGCTCAGTCTAAGG	AGGTGGGCTGACACAGACTT
BFRF3_qPCR	GGCGGATTTTCCAGACAGT	TGGGGAGATTATTCTGGTTCA
hMTR4_qPCR	TTGAAGGGTGTACACATGAGGT	TCCAACTCGTGGTTTAAGTGG
proEXT1_qPCR	TCTAATGGCTGCAGGGAAAC	TAGCTGGGACAGTTGGCAAT

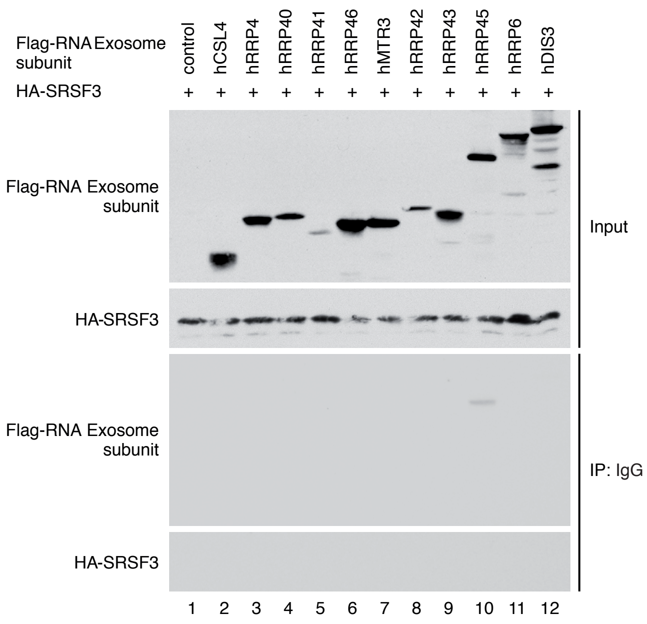

Supplementary Figure S1 (related to Figure 6)

SRSF3 does not interact with Protein G Sepharose beads. HA-SRSF3 was co-expressed with individual Flag epitope-tagged RNA exosome subunits in HeLa cells as indicated. The top panels show western blotting of 1/10 of total extract ('Input'), and the bottom panels show western blotting following immunoprecipitation with a control antibody (IP: IgG).

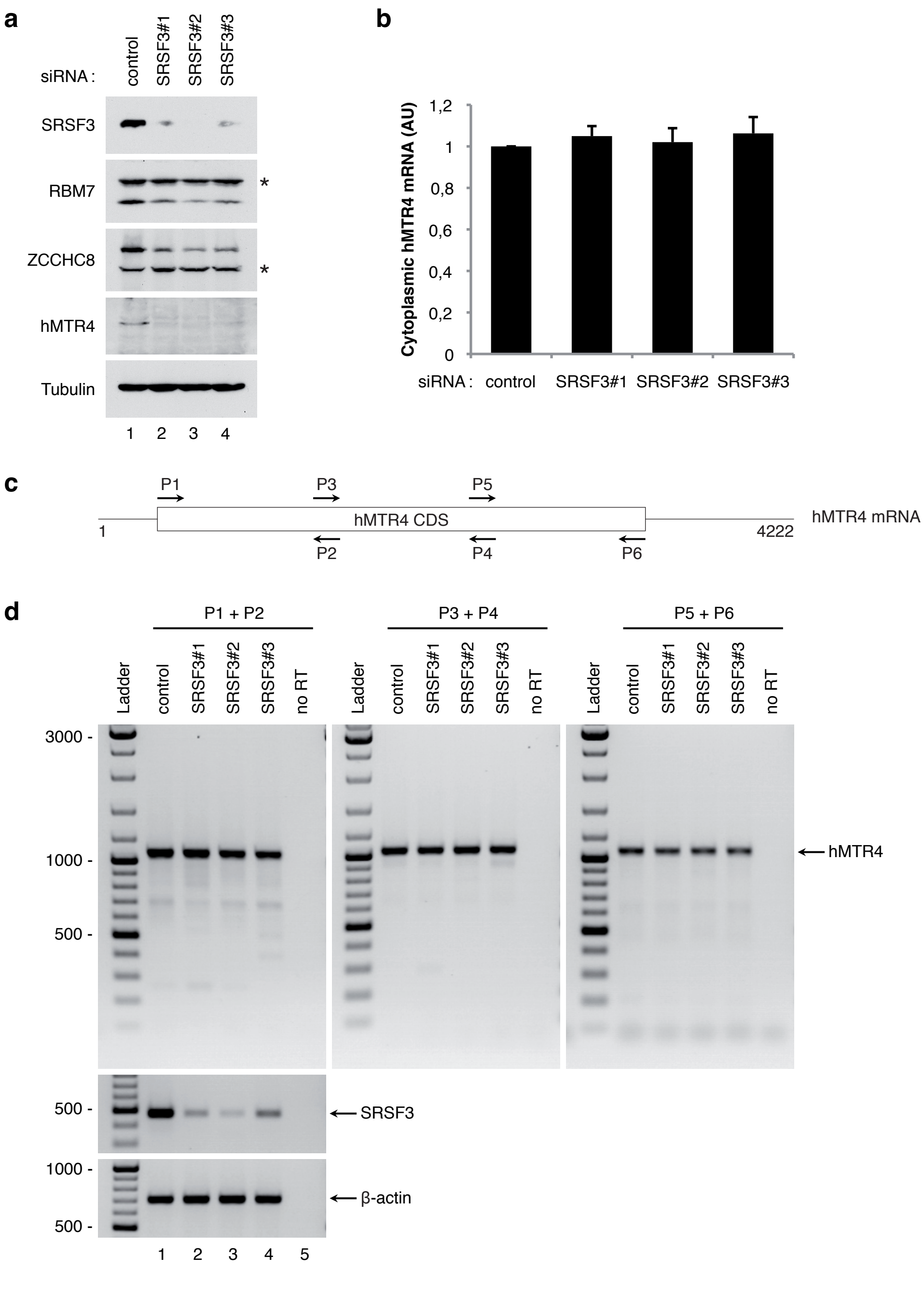

Supplementary Figure S2 (related to Figure 7)

Depletion of the splicing factor SRSF3 impacts the hMTR4 protein stability. (**a**) Western blotting analysis showing protein depletion upon treatments of HeLa cells with three different siRNAs directed against SRSF3. Anti-Tubulin antibody was used as a loading control. * indicates non-specific bands. (**b**) Quantification by RT-qPCR of the cytoplasmic hMTR4 mRNA from HeLa cells transfected with the indicated siRNAs. (**c**) Schematic representation of the hMTR4 mRNA. Black arrows represent the position of the primers used in the RT-PCR analysis. (**d**) RT-PCR analysis with three different sets of primers covering the full hMTR4 mRNA from HeLa cells transfected with the indicated siRNAs. The PCR products were loaded on a 1.5% agarose gel and visualized by ethidium bromide staining. The RT-PCR results were in the linear range of the PCR reaction.

Source data for Fig. 1a

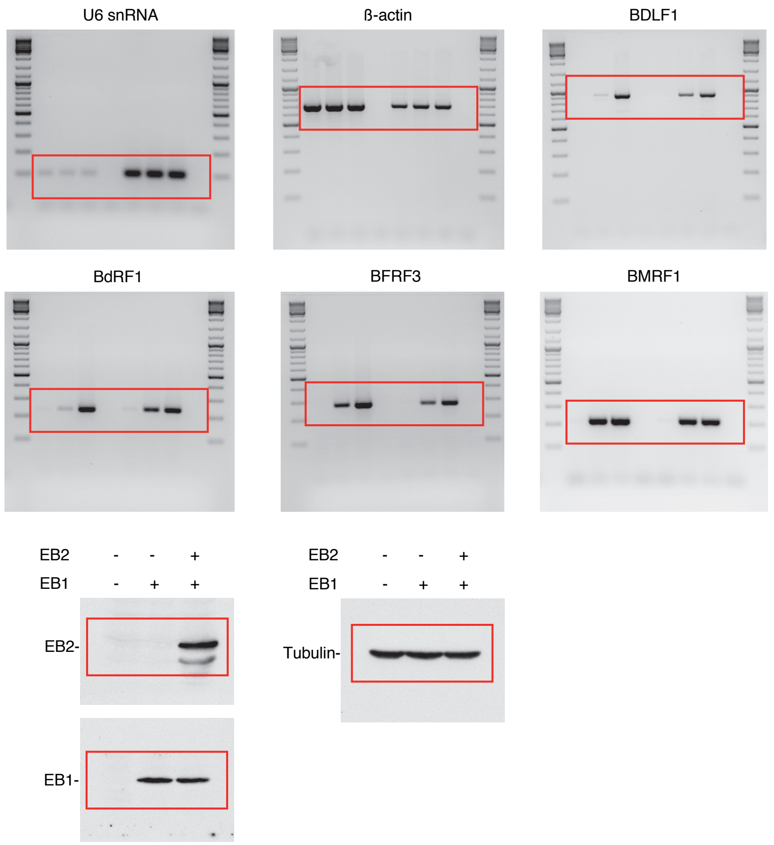

Source data for Fig. 2a

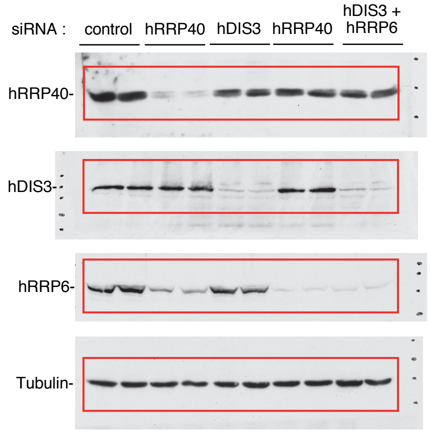

Source data for Fig. 2c

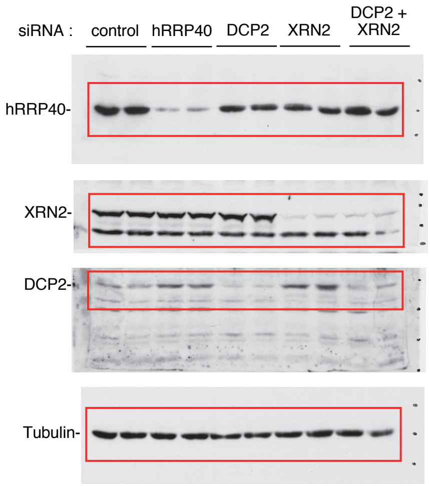

Source data for Fig. 3a

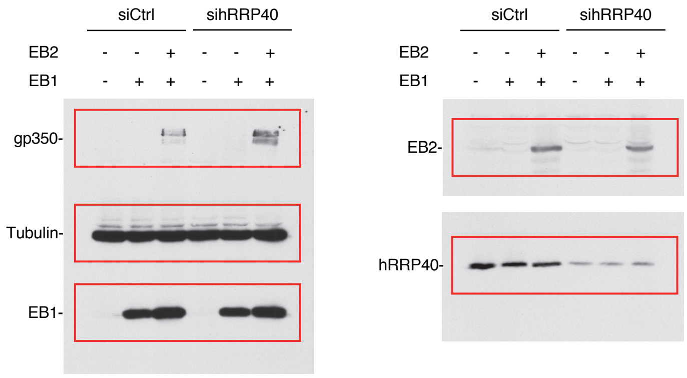

Source data for Fig. 4a

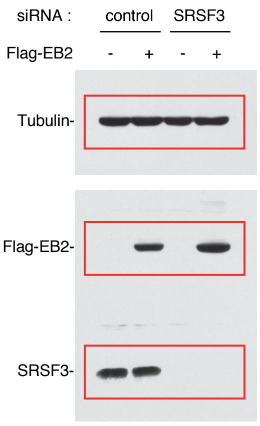

Source data for Fig. 4e

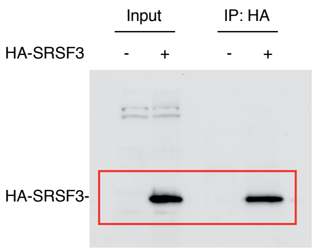

Supplementary Figure S3. Uncropped gels and blots.

Source data for Fig. 5a

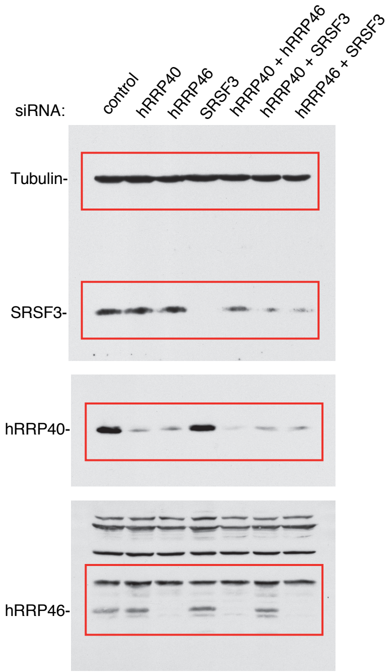

Source data for Fig. 6a

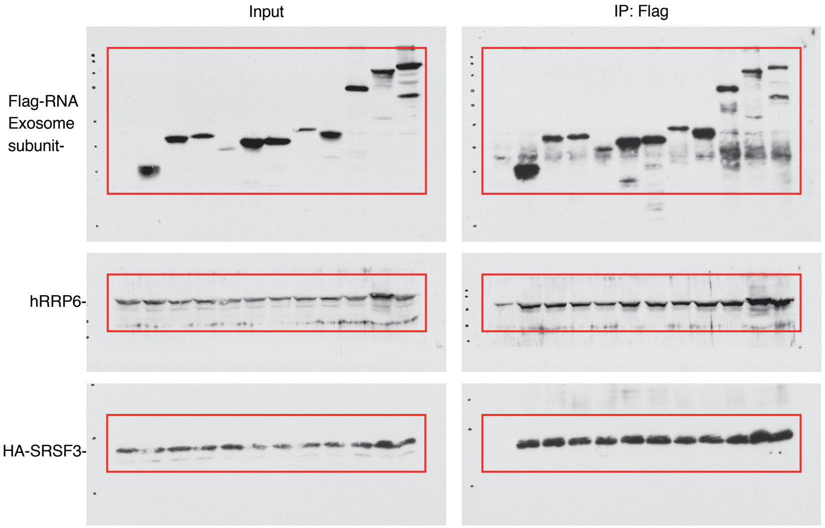

Source data for Fig. 6b

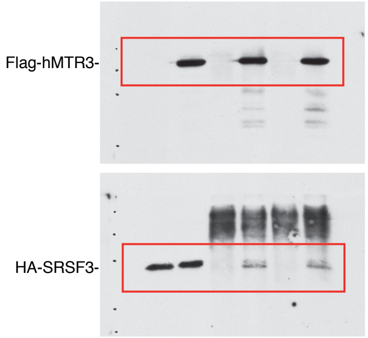

Source data for Fig. 6c

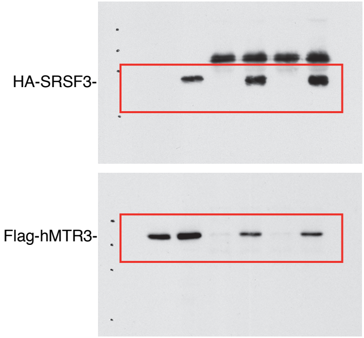

Source data for Fig. 6d

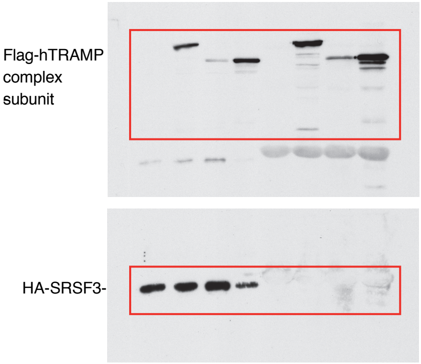

Source data for Fig. 6e

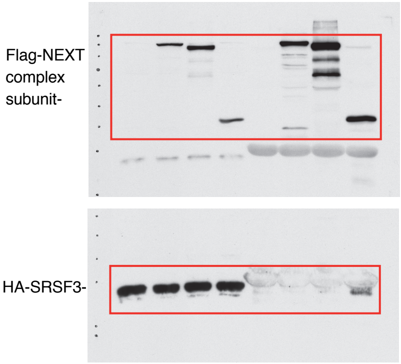

Source data for Fig. 6h

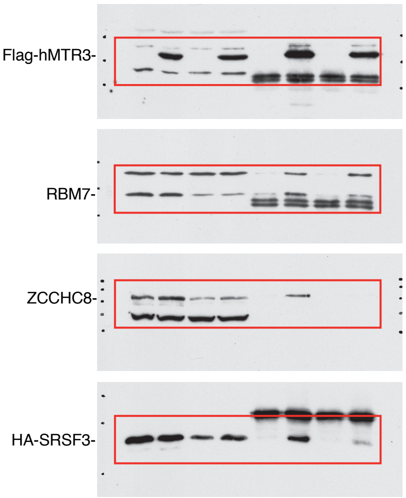

Source data for Fig. 6f

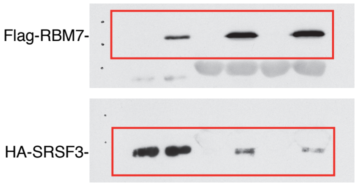

Source data for Fig. 6g

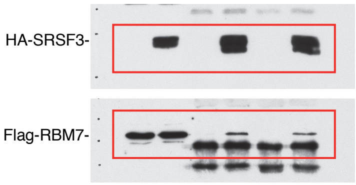

Supplementary Figure S3 (continued). Uncropped gels and blots.

Source data for Fig. 7a

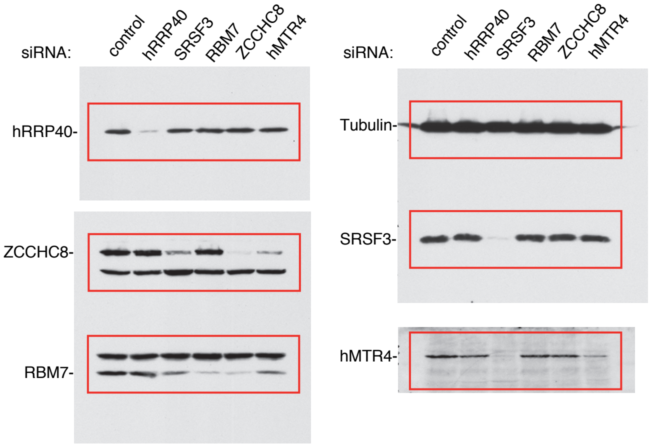

Source data for Fig. 7c

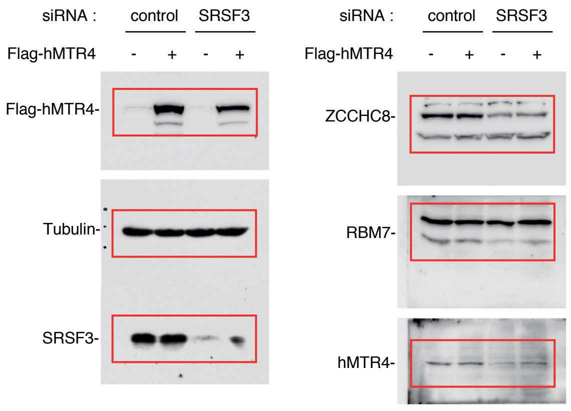

Source data for Fig. 7e

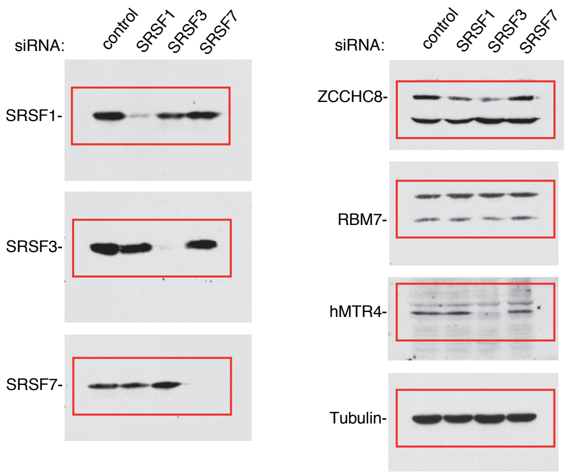

Source data for Fig. S1

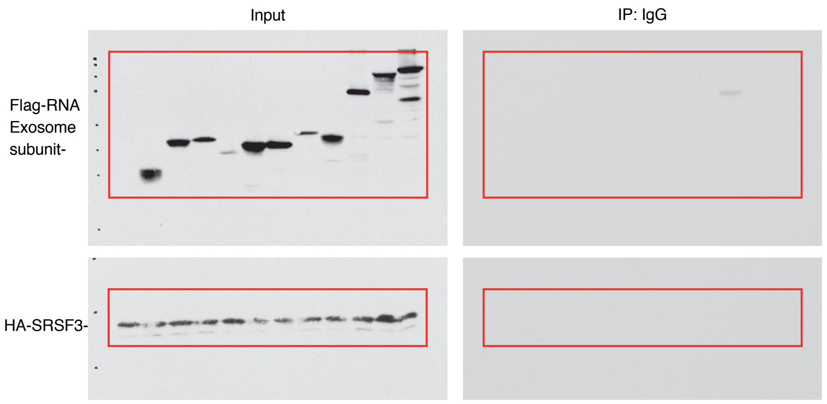

Source data for Fig. S2a

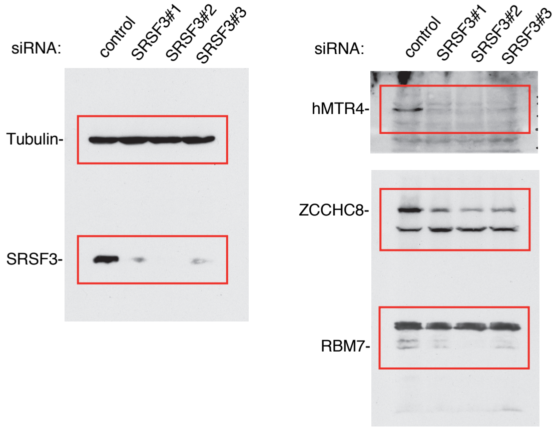

Source data for Fig. S2d

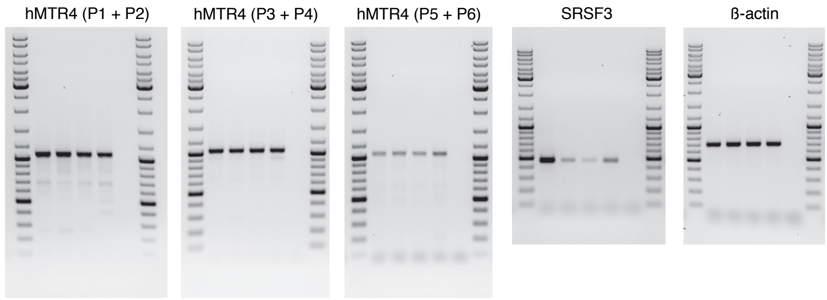

Supplementary Figure S3 (continued). Uncropped gels and blots.
